# Extracellular Small RNAs in Human Milk: Molecular Profiles, Stability and Fragment-Specific Responses in Cell-Based Assays

**DOI:** 10.3390/ncrna12010005

**Published:** 2026-02-09

**Authors:** Clara Claus, Carla Borini Etichetti, Bruno Costa, Julieta B. Grosso, Juan Pablo Tosar, Uciel Chorostecki, Silvana V. Spinelli

**Affiliations:** 1 Institute of Clinical and Experimental Immunology of Rosario, Suipacha 590, Rosario 2000, Argentina; claus@idicer-conicet.gob.ar (C.C.); grosso@idicer-conicet.gob.ar (J.B.G.); 2 Kresko RNAtech Corp., Wilmington, DE 19808, USA; borini@kreskornatech.com (C.B.E.); upchorostecki@uic.es (U.C.); 3 Institut Pasteur de Montevideo, Montevideo 11400, Uruguay; bcosta@pasteur.edu.uy (B.C.); jptosar@pasteur.edu.uy (J.P.T.); 4 School of Science, Universidad de la República, Montevideo 11800, Uruguay; 5 Department of Biomedical Sciences, Universitat Internacional de Catalunya, 08195 Sant Cugat del Vallès, Barcelona, Spain

**Keywords:** exRNA, cell survival, sRNA-seq, extracellular vesicles, tRNA fragments, human milk, RNA stability, non-vesicular RNA

## Abstract

**Background/Objectives:** Human milk is a complex biological fluid containing not only macro- and micronutrients but also diverse bioactive molecules, including extracellular RNAs. Although RNA has been detected in milk for decades, only a subset of RNA species has been characterized in detail, and abundant families such as tRNA-, yRNA-, and rRNA-derived fragments remain underexplored. This study aimed to define the composition, fragmentation patterns, stability, and exploratory functional activity of these highly abundant RNAs in human milk. **Methods:** We performed small RNA sequencing on skim milk samples and analyzed the resulting profiles in comparison with publicly available milk and biofluid datasets. RNA stability assays, Northern blotting, and RT-qPCR were conducted to validate RNA abundance and degradation kinetics. Extracellular vesicles (EVs) and non-vesicular fractions were analyzed to determine the subcellular distribution of RNA species. Exploratory functional assays using synthetic RNA fragments were carried out to assess their ability to modulate cellular responses in vitro. **Results:** Human milk was found to be highly enriched in small RNA fragments derived from tRNA, yRNA, and rRNA, dominated by a limited set of discrete sequences. These profiles were highly reproducible across independent datasets and distinct biofluids. Orthologal validation assays confirmed their abundance and stability, with RNA levels exceeding those of serum by over two orders of magnitude. Full-length transcripts were enriched in EVs, whereas shorter fragments predominated in the non-vesicular fraction. Synthetic milk-derived exRNAs showed detectable pro-survival activity under stress conditions in vitro. **Conclusions:** This study reveals that human milk carries a limited set of highly abundant stable sRNA molecules, primarily derived from tRNAs, yRNAs, and rRNAs. These findings provide new insights into the RNA cargo of human milk and offer preliminary evidence that selected sRNA fragments can modulate cellular stress responses in in vitro models.

## 1. Introduction

Evolution has played a key role in selecting the components of biological fluids to ensure their proper function. Milk is one of the most highly complex and heterogeneous biological fluids within biofluids, rich in macro- and micro-nutrients and bioactive compounds, like antimicrobial molecules, growth factors and antibodies. Human milk remains the gold standard for infant nutrition and plays important roles in the child’s first immune defense and gut health [1].

Different lines of evidence indicate that human milk contains high quantities of RNAs, which include long non-coding RNAs (lncRNAs), circular RNAs (cRNAs), and small RNAs (sRNA) [2,3]. sRNAs are non-coding RNA (ncRNA) typically between 18 and 200 nucleotides in length, produced by all human cells. These molecules regulate a wide range of biological processes—including cellular metabolism, development, proliferation, and gene expression—primarily through post-transcriptional mechanisms. They typically interfere with mRNA translation via RNA-RNA interactions, but can also influence transcription and other post-transcriptional pathways [4]. Among sRNAs, most studies have focused on microRNAs (miRNAs) and recent research suggests that abundant milk miRNA families have been implicated in modulating immune pathways, potentially contributing to the infant’s immune system development [5] and maturation [6]. These molecules are primarily encapsulated within extracellular vesicles and might be taken up by human intestinal cells and macrophages [6,7,8,9].

The results obtained in human milk align with data demonstrating the presence of extracellular small RNAs (exRNAs) in all human biofluids analyzed to date [3,10,11]. In 2020, Hulstaert et al. published the Human Biofluid RNA Atlas [3] where they described the complete extracellular transcriptome across a wide range of human biofluids (amniotic fluid, aqueous humor, ascites, bile, bronchial lavage fluid, human milk, cerebrospinal fluid, colostrum, gastric fluid, pancreatic cyst fluid, plasma, saliva, seminal fluid, serum, sputum, stool, synovial fluid, sweat, tear fluid and urine), using sRNA-seq to quantify different sRNA species. They demonstrated that it is technically feasible to generate extracellular transcriptome data from low-input biofluid samples, and, as a result, showed that the distribution of sRNA biotypes exhibits distinct patterns among the 20 different biofluids. Most of these sRNA originate from RNA polymerase I and III transcripts, tRNA, yRNA, and rRNA. Although these molecules are best known for their essential role in translation, experimental evidence has established that they are not merely end products but can also serve as sources of functional sRNAs [12,13]. In this context, tRNA-derived fragments (tRFs), also known as tRNA-derived RNAs (tDRs), result from the specific cleavage of precursor or mature tRNAs. Various studies have demonstrated that their expression is triggered by diverse stress stimuli, including oxidative stress, heat shock, and UV radiation [14,15]. However, they are also detected under non-stressed conditions [16]. Notably, stress stimuli induce the accumulation of tRNA fragments not as mere degradation products, but as functional molecules involved in gene regulation and cellular stress responses. The biogenesis and regulation of tDRs involve specific RNA nucleases, and cellular stress conditions and RNA modifications further influence these processes. This indicates that tDR generation is a well-regulated process, producing fragments with potentially significant biological functions rather than random degradation products [17]. yRNA is a very conserved class of small ncRNA ranging from 84 to 113 nucleotides long [18,19,20] and four species exist in humans: *RNY1*, *RNY3*, *RNY4* and *RNY5*; being *RNY1*, *RNY3* and *RNY4* highly expressed in many tissues [19,20,21]. yRNAs can be processed into yRNA fragments with distinct entities, often exhibiting modifications at the 5′ end [22]. Regarding rRNAs, while they have been extensively studied in the context of protein synthesis, they have not been as exciting to researchers because they are not yet directly involved in the regulation of gene expression, and their derived fragments have typically been considered waste products. However, similar to what has been shown for tRNA, rRNA-derived fragments accumulate in the extracellular space and their extracellular concentration increases in specific physiological conditions, such as abnormal cell death [15].

Although several RNA biotypes have been described in human milk, large-scale profiling across human biofluids indicates that extracellular small RNA populations are overwhelmingly dominated by fragments derived from tRNAs, rRNAs, and yRNAs [3,10,11]. These abundant RNA families remain comparatively underexamined in milk, where most studies have focused on miRNAs. This gap motivated us to concentrate on these high-abundance, least characterized components of the milk exRNA landscape.

To build a coherent framework connecting molecular identity with biological activity, our analyses followed a stepwise logic. We first profiled the sRNA composition of human milk and benchmarked it against public datasets. We then validated whether the most abundant sequences correspond to discrete molecular entities rather than random degradation fragments. Because their high abundance could reflect differential stability across biofluids, we next compared milk with human serum and evaluated RNA decay under physiological conditions. We then examined their biochemical compartmentalization within extracellular vesicles or soluble complexes, as this can influence stability and potential bioavailability. Finally, we assessed whether these abundant and stable RNAs exert measurable biological effects under cellular stress.

## 2. Results

### 2.1. Sequence-Centric Profiling Reveals a Limited Repertoire of Abundant sRNAs in Human Milk

To systematically characterise exRNAs in human milk, we conducted sRNA-seq on three milk samples from healthy donors. Our analysis followed a sequence-centric approach, focusing on sequences that map to curated collections of known human sRNAs, including tRNA, yRNA, rRNA, and miRNA. For comparative purposes, we incorporated sRNA-seq data from human milk samples available in the Human Biofluid RNA Atlas [3].

**Figure 1:**
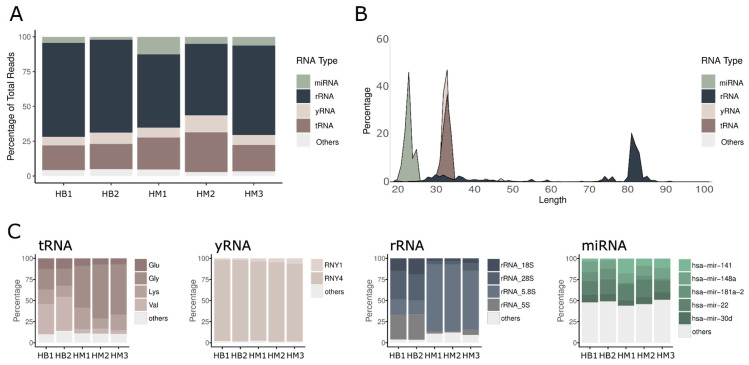
exRNA profiling in human skim milk. (**A**) Percentage distribution of sRNA biotypes in human skim milk samples collected for this study (HM1–3) and those described in the Human Biofluid RNA Atlas (HB1–2). (**B**) sRNA length distribution by biotype in the complete dataset. (**C**) Percentage distribution of the most abundant RNA fragments derived from tRNAs, rRNAs, and yRNAs in human skim milk. Categories with <5% average abundance across all samples were grouped as “others”.

Our analysis revealed that the most abundant exRNAs originated from transcripts of RNA polymerases I and III, such as tRNA, yRNA, and rRNA (Figure 1A and Supplementary Table S1). Among these, tRNAs represented 21.21 +/− 5% of the total sRNA content, yRNA made up 8.17 +/− 2% and rRNAs accounted for 60.50 +/− 8%. Interestingly, miRNA represents only a small proportion of the reads, with 6.07 ± 4%. This finding is consistent with the observations reported by Hulstaert et al., showing high levels of tRNA and rRNA in human biofluids [3].

We then examined the composition of exRNA within each category to identify abundant transcripts and assess their consistency. Among tRNAs, most sequences were 30–33 nucleotides (nt) in length, suggesting that they may represent halves of tRNAs resulting from cleavage of the mature transcript at the anticodon loop (Figure 1B). Notably, the majority of these sequences aligned with tRNA-Gly, tRNA-Val, tRNA-Glu, and tRNA-Lys, collectively comprising over 90% of this category across all samples, regardless of the dataset analyzed (Figure 1C and Supplementary Table S2). As for yRNA-related sequences, they were 30–32 nt in length and nearly all matched transcript 4 (Figure 1B,C and Supplementary Table S3). In contrast, sequences aligning to miRNAs (20–24 nt in length, consistent with their typical size), exhibited the highest diversity across the analyzed samples (Figure 1B,C and Supplementary Table S4). The most abundant miRNAs detected corresponded to members of the miR-22, miR-141, miR-148, miR-30, and miR-181 families, all previously reported in human milk [6]. These findings demonstrate strong concordance with established literature, reinforcing the reliability of our data.

When analyzing sequences aligning with rRNA, we observed significant discrepancies between datasets (Figure 1B,C and Supplementary Table S5). In our samples, most sequences measured 80–82 nt in length, whereas in the Atlas, fragments predominantly ranged from 30 to 40 nt (Supplementary Figure S1). This discrepancy directly correlates with the methodologies used for library preparation. In our experiments, we bypassed the agarose gel purification step commonly employed for miRNA studies to obtain a more comprehensive view of the abundant sRNAs in human milk. This protocol adaptation allowed us to identify novel species previously overlooked in other studies. Regarding sequence identity, we detected fragments within 5.8S rRNA being the most abundant in our samples. In contrast, in the milk samples from the Atlas, the most abundant are 28S and 5S rRNA-derived fragments (Figure 1C and Supplementary Table S5).

To evaluate whether this sRNA repertoire is preserved across lactation, we analyzed an independent set of nine human milk samples spanning colostrum, transitional milk, and mature milk up to 11 months postpartum. Across all samples, fragments derived from tRNAs, rRNAs, and yRNAs consistently accounted for the vast majority of mapped reads (94% ± 2%). An abundance-weighted intersection analysis further revealed a highly conserved core of dominant sequences shared across donors, with most high-abundance fragments present in all samples (Supplementary Figure S2). Although the late-lactation sample (11 months postpartum) exhibited slightly reduced overlap, its overall biotype distribution remained comparable. These results indicate that the major classes and most abundant species of milk-derived sRNAs exhibit remarkable stability across lactation.

To evaluate whether similar patterns extend to other human biofluids, we re-analyzed sRNA-seq data from the 20 biofluids in the Human Biofluid RNA Atlas, as well as 10 independent serum datasets processed in distinct laboratories. tDRs and yDRs displayed highly consistent profiles across fluids and datasets, whereas rDRs varied substantially depending on protocol-specific biases (Supplementary Figure S3).

Together, these results reveal that human milk—like most biofluids—is enriched in a restricted set of tRNA-, yRNA-, and rRNA-derived sequences that recurrently accumulate as highly abundant extracellular RNAs.

### 2.2. Orthogonal Validation Demonstrates That Abundant Milk sRNAs Correspond to Discrete tRNA-, yRNA-, and rRNA-Derived Fragments

Having defined the core set of abundant sRNAs in human milk, we next asked whether these molecules represent discrete RNA species or heterogeneous degradation products arising during sample handling or sequencing. To address this, we performed orthogonal validation assays capable of directly assessing transcript size and molecular identity.

**Figure 2:**
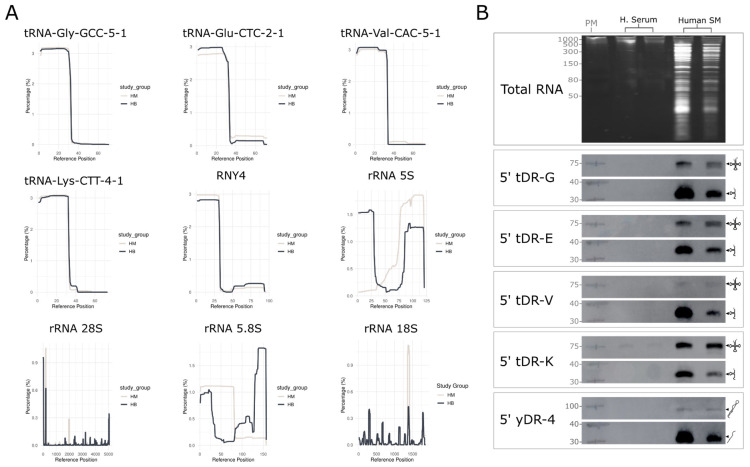
Fragment distribution and detection of abundant exRNA in milk. (**A**) Relative coverage across reference positions for specific RNA types in human skim milk samples from this study (HM) and the Atlas dataset (HB) The *x*-axis represents the reference position on the RNA type, and the *y*-axis represents the percentage of coverage at each position. (**B**) Northern blot of RNA purified from equal volumes (200 uL) of human serum and human skim milk samples. Specific probes were used to detect the following RNA fragments: 5′ tDR-G, 5′ tDR-E, 5′ tDR-V, 5′ tDR-K, and 5′ yDR-4. Exposure times of 3–4 min were used. Abbreviations: PM, prestained marker; H. Serum, human serum; Human SM, human skim milk.

To validate the molecular identity of abundant exRNAs, we mapped sRNA-seq reads across their source transcripts and generated coverage plots for tRNAs, yRNAs, and rRNAs (Figure 2A). Our analysis shows that abundant sequences detected in milk derived from tRNA-Gly, tRNA Glu, tRNA Val and tRNA Lys correspond to 5′ tRNA halves. Similarly, for yRNA-derived fragments, the most abundant fragment corresponds to the 5′ region of transcript 4 (Figure 2A). As anticipated, sequence alignments to rRNA revealed distinct patterns depending on the dataset analyzed. Notably, the coverage of these rRNA transcripts was not uniform, with certain discrete regions being overrepresented (rRNA 5S, 28S, 5.8S and 18S, Figure 2A).

Northern blot assays with probes targeting the 5′ regions of tRNA Glu, Gly, Val, and Lys (5′ tDR-G, 5′ tDR-E, 5′ tDR-V and 5′ tDR-K, respectively), as well as yRNA4 (5′ yDR-4), validated the presence of ~30 nt tRNA fragments (Figure 2B), consistent with those identified by sRNA-seq in human milk. Notably, full-length transcript bands were detected in milk samples for all analyzed tRNAs and for yRNA4 (Figure 2B). These species were barely or not detectable in serum, reflecting the lower RNA content of this fluid [3].

**Figure 3:**
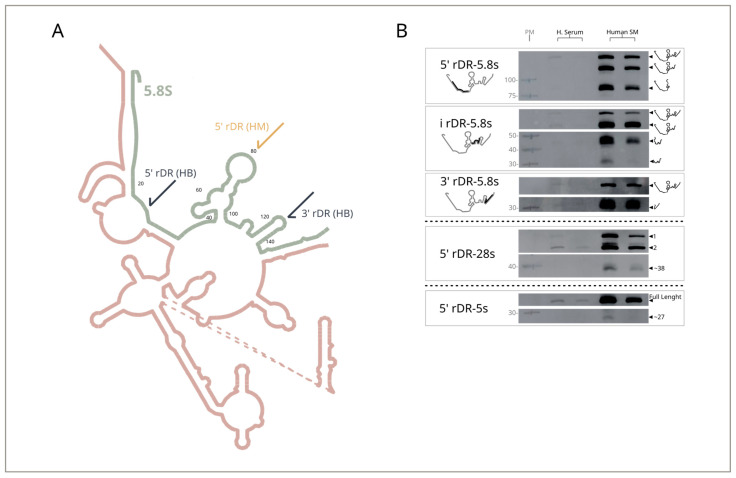
Detection of sRNA fragments derived from ribosomal RNA. (**A**) Schematic representation of the secondary structure of the human 60S ribosomal subunit [23]. Lines indicate cleavage sites marking the beginning or end of the 3′ and 5′ fragments derived from rRNA 5.8S, respectively. Cleavage sites detected in our sequencing experiments are shown in yellow, while those from the Atlas are shown in black. (**B**) Northern blots detecting 5′, 3′, and internal fragments of 5.8S rRNA; additionally, fragments from 5′-end of 28S and 5S rRNAs in serum and skim milk. SYBR staining of total RNA is shown in Figure 2B and is not repeated here. Exposure times of 3–4 min were used. Abbreviations: PM, Prestained Marker; H. Serum, Human Serum; Human SM, Human Skim Milk.

Northern blot assays were also employed to investigate rRNA-derived fragments further and determine whether specific sequences accumulate in human milk. As a representative example, we analyzed fragments of 5.8S rRNA, the most abundant rRNA in our dataset. The schematic in Figure 3A shows how the fragmentation pattern identified by sequencing data correlates with exposed regions in the predicted ribosome structure. By using region-specific probes, we confirmed that fragments derived from this rRNA accumulate in human milk, with the ~80 nt fragment identified in our sequencing experiments and two short fragments from the Atlas being found in high abundance. Also, a full-length transcript band and other higher molecular weight cutting intermediates were detected (Figure 3B).

To complement the characterization of rRNA-derived fragments, we also analyzed the 5′ region of 28S rRNA and the 5′ region of 5S rRNA, as these represent the major rRNAs in the samples from the Human Biofluid RNA Atlas (Figure 1C). In these cases, we observed that human milk accumulates a 5′ fragment derived from 28S rRNA ≤ 40 nt (5′ rDR-28s, Figure 3B), along with other high-molecular-weight species. This observation aligns with findings in the literature [24]. For 5S rRNA, the full-length transcript was the predominant species detected, accompanied by several lower molecular weight fragments. The 25-nt fragment identified by sequencing was also present, though at much lower abundance (5′ rDR-5s, Figure 3B).

These findings indicate that, while useful, sequencing provides incomplete or misleading information about abundant sRNAs in biofluids. In milk samples, our Northern blotting experiments confirmed the presence of discrete tRNA- and yRNA-derived fragments and further contributed to the characterization of rRNA-derived fragments.

### 2.3. exRNAs Are Found in Higher Levels and Exhibit Greater Stability in Milk Compared to Serum

The accumulation of well-defined tDRs, yDRs, and rDRs in milk prompted us to ask why these molecules are so enriched in this biofluid. Their high abundance could reflect active secretion, increased stability, or both. Because serum is an RNase-rich environment where extracellular RNAs are rapidly degraded, we next benchmarked milk against human serum and directly evaluated transcript stability.

**Figure 4:**
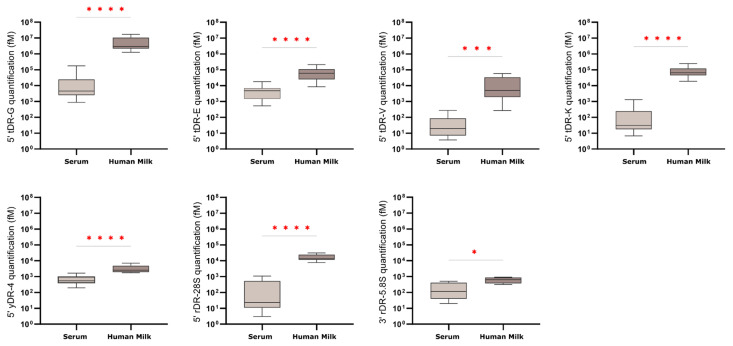
Quantification of specific RNA fragments by SLO RT-qPCR. Expression levels of 5′ tDR-G, 5′ tDR-E, 5′ tDR-V, 5′ tDR-K, 5′ yDR-4, 5′ rDR-28S, and 3′ rDR-5.8S were measured by SLO RT-qPCR in RNA from human serum (n = 14) and human skim milk (n = 8). Data are shown as mean ± SEM. Quantification used standard curves from synthetic RNA oligos. Statistical analysis was determined using a two-tailed unpaired Mann–Whitney U test with α = 0.05; significance levels indicated as *p* < 0.05 (*), *p* < 0.001 (***), *p* < 0.0001 (****).

To further validate the presence of the sequences identified in the previous section, we employed the highly sensitive RT-qPCR technique and expanded the analysis to a larger cohort of samples (human milk, n = 8; serum, n = 14). For these experiments, we quantified the four 5′ tRNA halves derived from tRNA Gly, Glu, Val and Lys; and the 5′ fragment of yRNA-4. As for rRNA-derived fragments, we selected 5′ 28S and 3′ 5.8S, as structural analyses suggest these sequences are base-paired within the secondary structure of the 60S ribosomal subunit—an arrangement that may contribute to their stability and accumulation in biofluids (Figure 3A). All tested RNAs were detected in both fluids, but their levels were, on average, nearly two orders of magnitude higher in milk (Figure 4). This mirrors Northern blot results and confirms that serum contains substantially lower exRNA content. The only exception was the 3′ fragment of 5.8S rRNA, which showed equally low levels in both serum and milk when measured by RT-qPCR—contrary to the results obtained via Northern blotting. This discrepancy indicates that RT-qPCR is not a reliable method for detecting this fragment, leading to its exclusion from future studies (Figure 4).

**Figure 5:**
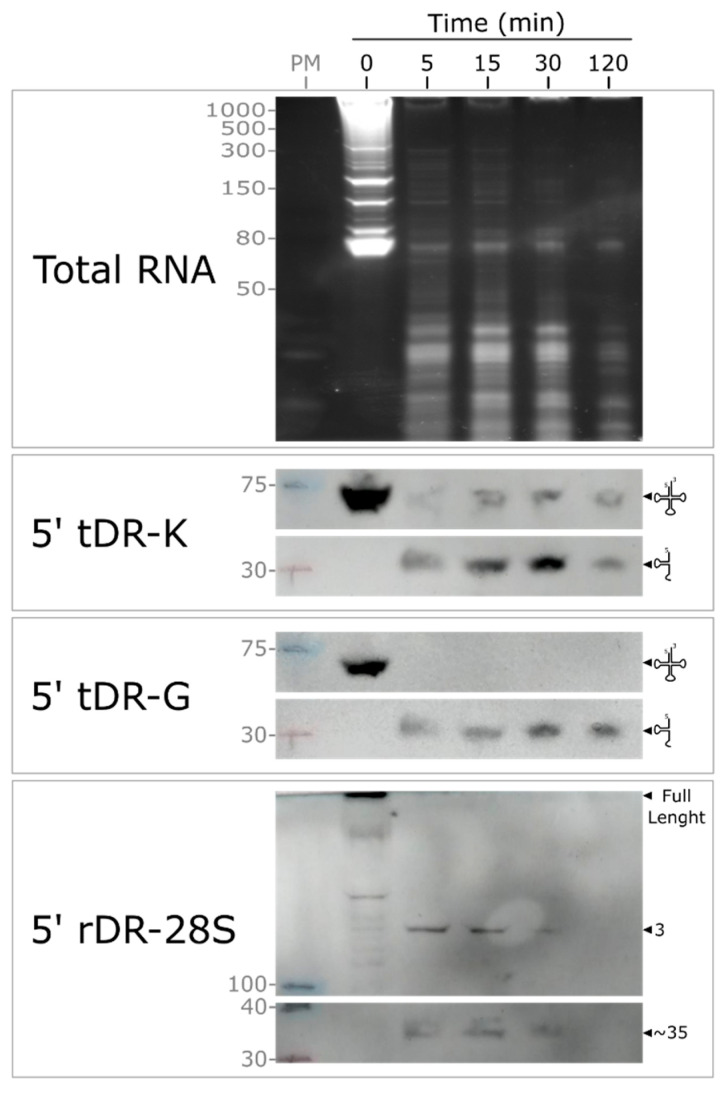
exRNA stability in human milk. Northern blots detecting 5′ tDR-G, 5′ tDR-K, and 5′ rDR-28S after incubation of purified human-cell RNA in skim milk for 5, 15, 30, and 120 min. Exposure times of 3–4 min were used. Abbreviation: PM, prestained marker.

To assess stability under physiological conditions, we performed RNA decay assays by spiking purified cellular RNA into fresh milk and monitoring fragment persistence at 37 °C (Figure 5). Full-length tRNA-Lys remained detectable for ≥2 h, and a stable 5′ tRNA-Gly fragment appeared within seconds and persisted throughout the experiment. The 5′ 28S fragment also persisted for ≥30 min. These profiles contrast with serum, where similar RNA species degrade within minutes [24].

Together, these data indicate that milk constitutes a protective biochemical niche that supports the accumulation and long-term persistence of extracellular RNAs.

### 2.4. Differential Distribution of Milk exRNA Within Vesicles and Soluble Fractions

Because RNA stability in extracellular environments is strongly influenced by biochemical compartmentalization—either through encapsulation within extracellular vesicles (EVs) or association with soluble protein complexes—we next examined how abundant milk-derived sRNAs partition across these compartments. Our goal in these experiments was not to achieve exhaustive EV purification, but rather to determine whether the most abundant sRNA species detected in milk reside primarily in vesicular or non-vesicular fractions. Human milk samples did not provide the milliliter-scale input required to perform ultracentrifugation (UC) and size-exclusion chromatography (SEC) in parallel while maintaining sufficient RNA material for Northern blotting and RT-qPCR analyses. Therefore, and because bovine milk exhibits an sRNA profile highly similar to human milk (Supplementary Figures S4 and S5), fractionation experiments were conducted using bovine skim milk as an experimentally tractable system for compartmentalization studies.

**Figure 6:**
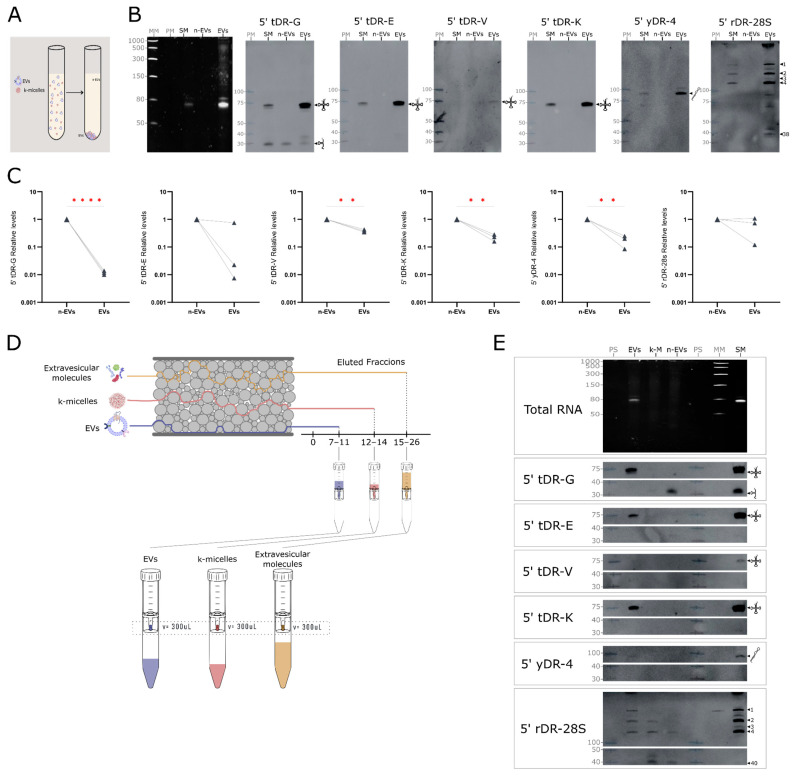
Milk exRNA distribution between vesicular and non-vesicular fractions. (**A**) Schematic representation of the ultracentrifugation fractionation workflow. (**B**) Northern blot detection of 5′ tDR-G, 5′ tDR-E, 5′ tDR-V, 5′ tDR-K, 5′ yDR-4, and 5′ rDR-28S in EV and n-EV fractions. Exposure times of 3–4 min were used. (**C**) RT-qPCR quantification of milk exRNA candidates. Quantification was performed using standard curves from synthetic RNA oligonucleotides. exRNA levels within each fraction were determined by normalizing to initial volume. Data are shown as paired individual values (n = 3 biologically independent replicates). Statistical analysis: two-tailed paired *t*-test; significance indicated as *p* < 0.01 (**), *p* < 0.0001 (****). (**D**) Schematic of the size-exclusion chromatography (SEC) workflow. (**E**) Northern blot detection of 5′ tDR-G, 5′ tDR-E, 5′ tDR-V, 5′ tDR-K, 5′ yDR-4, and 5′ rDR-28S in SEC-eluted fractions. Abbreviations: EVs, extracellular vesicles; k-M, micellar fraction; n-EVs, non-vesicular fraction; SM, skim milk; PS, prestained marker, MM molecular marker.

Ultracentrifugation generated a broad separation between vesicular (EV-enriched) and non-vesicular (n-EV) material (Figure 6A). Upon concentrating the EV-enriched fraction, Northern blot analysis revealed the presence of full-length tRNAs and yRNAs, along with a range of 28S rRNA-derived fragments (Figure 6B). In contrast, only 5′ tRNA halves derived from Gly tRNAs were detectable among the sRNA fragments. A 200 μL aliquot of the n-EV fraction (1/50th of the total volume) was also analyzed, where only Gly-derived fragments were detectable by northern blot (5′ tDR-G, Figure 6B). Since the sensitivity of Northern blotting was insufficient to detect sRNA fragments in most n-EV samples, RT-qPCR was employed for more sensitive detection. RT-qPCR analysis showed that 5′ tRNA halves derived from Gly, Glu, Val, and Lys tRNAs, as well as yRNA-4, were enriched in the n-EV fraction, while 5′ 28S rRNA fragments were equally detected in both EV and n-EV fractions (Figure 6C). Because UC co-pellets casein micelles together with EVs, the presence of rRNA-derived fragments in UC pellets could reflect either vesicular association or micelle co-sedimentation. To resolve this ambiguity, we incorporated SEC as an orthogonal method capable of separating EVs, casein micelles, and soluble proteins with high biochemical resolution (Figure 6D and Supplementary Figure S6).

Our results are shown in Figure 6E and indicate that EVs are predominantly enriched in full-length tRNA and yRNA transcripts, as well as high-molecular-weight rRNA fragments. In contrast, all sRNA fragments detected were found in the n-EV fraction. Among these, 5′ tRNA-Gly and 28S rRNA fragments showed the strongest signals, while 5′ tRNA-Glu and 5′ tRNA-Lys were only weakly detected. Notably, the 5′ 28S rRNA fragment also showed association with casein micelles (5′ rDR 28s, Figure 6E). This observation refines the interpretation of qRT-PCR data obtained following ultracentrifugation, which detected 5′ 28S rRNA in both EV and n-EV fractions. As ultracentrifugation does not discriminate between extracellular vesicles and casein micelles—which co-pellet under these conditions—the presence of micelles likely results in an overestimation of RNA species specifically associated with EVs, explaining its apparent association with UC pellets and clarifying that its distribution reflects micelle co-sedimentation rather than genuine EV enrichment.

Taken together, UC and SEC results in bovine samples converge on a coherent compartmentalization pattern: longer RNA species such as full-length tRNAs, full-length yRNAs, and high-molecular-weight rRNA fragments are preferentially associated with EVs, whereas shorter sRNA fragments—including the abundant 5′ tRNA halves and 5′ rRNA-derived fragments—are predominantly non-vesicular, residing either freely in solution or associated with soluble protein complexes such as casein micelles.

### 2.5. Milk exRNAs Contribute to Cell Survival Mechanisms

The identification of abundant, discrete, stable, and compartmentalized RNA species in milk raises the possibility that they contribute to functional responses in recipient cells. We therefore examined whether milk exRNA retains the ability to modulate cellular responses in vitro, using selected synthetic RNA fragments.

Given extensive evidence that tDRs are produced under cellular stress and can enhance cell survival pathways [12,13,14,15,16,25,26], we next evaluated whether these abundant exRNAs exert a protective effect on cells exposed to nutritional stress. Importantly, these assays were designed as reductionist, proof-of-principle experiments to evaluate intracellular activity rather than to model physiological exposure. Specifically, we assessed cell viability in a panel of five cell lines cultured under nutrient deprivation by limiting FBS to 0.1%. The cells were treated with or without synthetic RNA oligonucleotides that mimic representative sequences of 5′ tRNA halves derived from tRNA Gly, Glu, Val and Lys; and the 5′ fragments of yRNA4 and 5′ rRNA 28S (Supplementary Figure S7).

**Figure 7:**
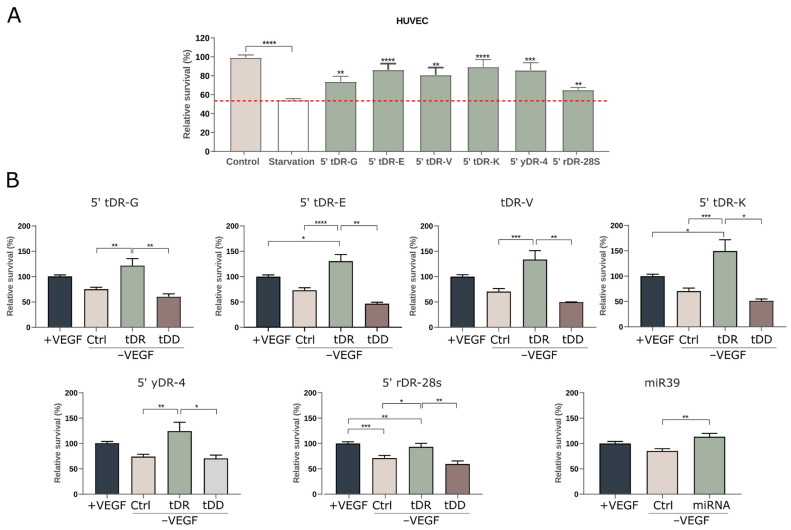
Functional effects of ex-sRNA candidates on HUVEC survival under stress conditions. (**A**) Effect of starvation and exRNA candidates on HUVEC cell survival. The tested exRNA candidates include 5′ tDR-G, 5′ tDR-E, 5′ tDR-V, 5′ tDR-K, 5′ yDR-4, and 5′ rDR-28S. Statistical significance was determined using a one-tailed *t*-test (Starvation vs. Treated), with significance levels indicated as *p* < 0.01 (**), *p* < 0.001 (***), and *p* < 0.0001 (****). (**B**) Effect of VEGF deprivation and exRNA candidates on 0.1% FBS cultured-HUVEC cell survival. tDD accounts to DNA oligonucleotides containing predominantly identical sequence motifs to the exRNA candidate tested within each graph. Statistical significance was determined using ANOVA, with significance levels indicated as *p* < 0.05 (*), *p* < 0.01 (**), *p* < 0.001 (***), and *p* < 0.0001 (****).

The effect of nutritional deprivation on cell viability was evaluated in HUVEC, HEK293, HaCaT, THP-1, and Caco-2 cell lines. Notably, serum deprivation did not affect THP-1 viability (Supplementary Figure S8A), likely due to PMA-induced terminal differentiation and lack of proliferation; therefore, this cell line was excluded from subsequent analyses. Among the remaining four cell types, all tested RNAs promoted survival specifically in vascular endothelial cells (HUVECs) (Figure 7A), but had no effect on the viability of HaCaT, Caco-2, or HEK293 cells (Supplementary Figure S8B–D).

To further investigate the impact of stress on vascular cells, we cultured HUVEC cells under more restrictive growth conditions by removing vascular endothelial growth factor (VEGF), an essential growth factor for this cell line. Our results showed that cells treated with synthetic sRNAs exhibited increased viability, in most cases, often doubling control values and surpassing the effects of VEGF alone (Figure 7B). Intriguingly, all tested sRNAs demonstrated pro-survival activity, in contrast to DNA oligonucleotides with largely identical sequence motifs. Similar results were observed with *C. elegans* miR-39, a standard spike-in RNA that is not found in humans, not present in milk, and not normally ingested. Its inclusion served solely as a non-endogenous control, further supporting that the observed effect operates through a sequence-independent pathway.

**Figure 8:**
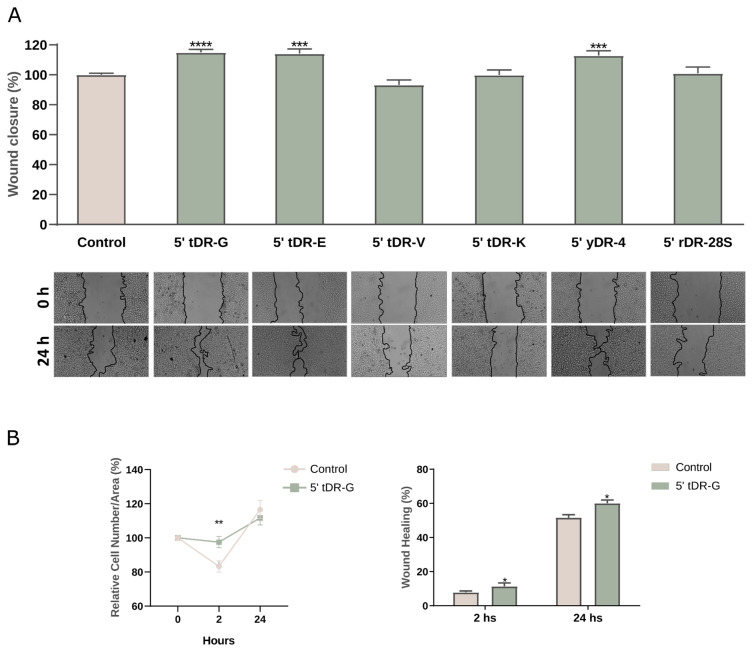
exRNAs modulate HaCat cell wound closure and survival. (**A**) Wound closure (%) in HaCaT cells 24 h post-scratch under treatment with 5′ tDR-G, 5′ tDR-E, 5′ tDR-V, 5′ tDR-K, 5′ yDR-4, or 5′ rDR-28S. Statistical significance was determined using one-tailed *t*-test (treated vs. control), with significance levels indicated as *p* < 0.001 (***), and *p* < 0.0001 (****). Representative micrographs shown below. (**B**) Effect of 5′ tDR-G on HaCat cell survival and wound closure. The left panel shows the number of HaCat cells per area after 2 and 24 h post-wound closure. The right panel illustrates the wound closure rate at 2 and 24 h in response to 5′ tDR-G treatment. Statistical significance was determined using one-tailed *t*-test, with *p* < 0.05 (*), *p* < 0.01 (**).

The differences observed between HUVECs and the other cell lines may be due to their primary origin, which makes them more sensitive than the immortalized lines tested. To further test our hypothesis, we conducted scratch assays in keratinocytes as an alternative injury-associated stress model. The scratch mimics physical injury, creating a context in which cells at the wound edges experience mechanical stress. Interestingly, we found that among the synthetic oligonucleotides tested, those homologous to the 5′ ends of tRNA Gly, tRNA Glu, and yRNA4 significantly accelerated wound closure compared to control conditions (Figure 8A).

To investigate this effect, additional experiments were conducted with the oligonucleotide 5′ tDR-G, quantifying the percentage of wound closure at different time points and assessing the cell count per unit area. Under normal conditions, a decline in cell count was observed during the initial hours post-scratch, followed by recovery and an increase around the 24-h mark. However, the addition of 5′ tDR-G prevented significantly the initial decline in cell count (Figure 8B). These findings are consistent with observations in HUVEC cells, suggesting a role for extracellular sRNAs in promoting cell survival. To complement the in-vitro functional assays, we performed exploratory mRNA-seq in epithelial cells subjected to scratch-induced stress with or without synthetic 5′ tDR-G. A short 2-h exposure was selected to capture early transcriptional responses. The analysis revealed only a limited set of differentially expressed transcripts (Supplementary Table S6), indicating that the treatment elicits a modest transcriptional footprint. This pattern is consistent with the notion that small RNA fragments predominantly act through post-transcriptional or translational mechanisms rather than through broad transcriptional remodeling [27].

## 3. Discussion

In this work, we provide an expanded view of the small-RNA landscape of human milk by shifting the analytical focus beyond miRNAs. Our sequence-centric profiling reveals that the extracellular RNA repertoire of milk is dominated by a restricted set of highly abundant fragments derived from tRNAs, rRNAs, and yRNAs—RNA classes that are widespread across human biofluids yet remain comparatively understudied in this context. Analysis of an independent cohort spanning colostrum to late lactation further shows that these fragments consistently constitute the major sRNA components across stages, supporting the existence of a robust core exRNA signature in human milk.

Notably, the predominance of sRNA fragments ranging from 18 to 40 nt in length suggests that these molecules may play regulatory roles in extracellular fluids. Their small size and structural stability make them uniquely suited to function as molecular messengers in diverse biological processes [28]. The observed consistency in the abundance of specific 5′ tRNAs fragments of tRNA Glu, Gly, Val, and Lys across different samples reinforces the notion that these molecules are not merely degradation products but may represent functional entities with physiological roles. Previous studies have suggested that tRNA-derived fragments can act as post-transcriptional regulators, influencing gene expression, immune responses, and metabolic pathways [29]. Additionally, earlier studies have shown that certain tRFs can interact with ribosomal subunits or RNA-binding proteins, modulating translational efficiency and cellular stress responses [30,31]. These mechanisms suggest that the high abundance of tRNA-derived fragments in milk may have a functional significance beyond simple RNA turnover.

Regarding rRNA-derived fragments, the existing data are more challenging to integrate, as diverse methodologies have provided complementary insights. These observations highlight the need for a more systematic exploration of rRNA-derived fragments in biofluids and the development of robust analytical tools to precisely characterize them. Importantly, our findings demonstrate that fragments derived from these transcripts are present at high concentrations in milk, revealing a previously understudied class of RNA fragments that merits further investigation. Similarly, yRNA4-derived fragments represent another promising area of research, with limited evidence suggesting their significant abundance in platelets and a potential involvement in cardioprotective mechanisms [32].

Our findings highlight key discrepancies between sequencing-based RNA profiling and Northern blot validation. While sRNA-seq effectively identified ~30 nt tRNA fragments in human milk, Northern blot analysis also detected full-length tRNAs, which were nearly absent in the sequencing data. Similarly, for rRNA-derived fragments, the sequencing data suggested specific fragmentation patterns, which were largely confirmed by Northern blot, particularly for the ~80 nt 5.8S rRNA fragment. However, Northern blot detected full-length rRNAs and high-molecular-weight intermediates, which were not prominent in sequencing datasets, in milk samples. These discrepancies are consistent with well-documented limitations of ligation- and reverse-transcription–based sRNA-seq methods, which under-represent full-length tRNAs and rRNAs due to extensive nucleotide modifications and highly structured regions that impede adapter ligation and cause RT stalling. This bias is widely recognized across sequencing platforms [33]. Northern blotting, which bypasses these biochemical constraints, therefore captures intact species that are not efficiently represented in sequencing datasets.

Furthermore, compared to other biofluids such as serum and plasma, milk shows a marked enrichment of these discrete RNA fragments, which may be attributed to various matrix-associated protective factors (e.g., protein or lipid association, EV encapsulation, and overall reduced RNase accessibility). Our decay assays demonstrate that exogenous tRNA and rRNA fragments have prolonged half-lives in human milk relative to serum and most other human biofluids studied [24]. This enhanced stability may confer an evolutionary advantage by preserving these RNA species for potential uptake by neonatal cells during breastfeeding. The protective environment of human milk may thus facilitate the delivery of functional RNA molecules to the infant, supporting early development. Recent in vivo evidence in neonates and a porcine model indicates that dietary milk miRNAs can withstand gastrointestinal passage, be taken up by intestinal cells, and show initial signs of functional engagement [34]. Our findings are consistent with previous reports showing that miRNAs and other sRNA species are resistant to harsh conditions and can survive digestion [7,8,35].

Interestingly, the RNA species detected in milk show remarkable parallels with profiles previously observed in conditioned media from human cell cultures treated with RNase inhibitors. For instance, the presence of full-length 5.8S rRNA and a defined ~80 nt 5′ fragment, confirmed here by Northern blot, mirrors findings in extracellular ribosomal fractions described by Tosar et al. (2020) [29], where a similar cleavage pattern was observed at position 80–83. This raises the intriguing possibility that extracellular ribosomes—so far not described in milk—may also be present in this biofluid.

The characterization of exRNAs also revealed distinct distribution patterns within EV and n-EV fractions of milk. Our findings in bovine samples indicate that while larger RNA fragments are predominantly found within EVs, small exRNA are enriched in the n-EV fraction. A similar distribution of full-length and fragmented tRNAs between EV and n-EV fractions has also been reported in cell culture systems [29], supporting the biological relevance of our findings. This differential distribution suggests that various transport mechanisms may be at play, influencing how these RNAs interact with recipient cells. Recent studies have characterized the sRNA cargo within EVs from human milk, demonstrating that they can induce changes in gene expression and may play roles in endocrine signaling and immune modulation [36].

n-EV RNAs have been historically overlooked due to technical challenges and the belief that their lack of membrane protection makes them unsuitable for intercellular communication. Recent evidence, however, shows that some n-EV RNAs are stable in extracellular environments, allowing them to potentially act as functional signaling molecules [37,38]. Recent studies have demonstrated that naked RNAs can be internalized and sensed by epithelial cells via endocytic pathways under low-RNase conditions [39], supporting the hypothesis that non-vesicular exRNAs in milk may be taken up by infant gut cells and exert biological functions even in the absence of vesicular protection. This insight provides a mechanistic rationale for the potential activity of milk-derived RNAs in early development.

Comparative analysis of human and bovine milk revealed highly similar exRNA profiles, primarily composed of discrete fragments derived from tRNAs, rRNAs, and yRNAs. These results are consistent with data from the Human Biofluid RNA Atlas and support the notion that these RNA signatures are evolutionarily conserved across species and biofluids. Previous studies have shown that such fragments are generated in response to cellular stress and may play roles in promoting cell survival [12,13,14,15,16,25,26]. To assess the potential bioactive activity of milk-derived exRNAs, we subjected human cell lines to nutritional stress and treated them with synthetic oligonucleotides matching selected RNA candidates. Interestingly, all tested RNAs enhanced cell viability in HUVEC cells, suggesting a protective mechanism that may act independently of specific sequence features. However, this effect was not observed in the other cell types tested, and further studies in keratinocytes showed that only three of the RNAs tested accelerated wound closure in scratch assays. This variability could be attributed to the limitations associated with using immortalized cell lines and synthetic RNAs, since our functional assays relied on synthetic RNAs rather than naturally isolated exRNAs. This experimental design overlooks critical physiological factors such as RNA modifications and ribonucleoprotein complex-mediated protection, both of which are known to influence stability, uptake, and biological activity. Importantly, while our functional assays do not model the well-documented reparative properties of human milk [40,41,42], they demonstrate that abundant milk sRNAs can modulate cellular stress responses in vitro. Whether such activity plays any physiological role remains unknown and merits future study.

The results presented above provide preliminary evidence for a potential regulatory role of milk-derived exRNAs. Given the central role of human milk in neonatal nutrition, our findings support the hypothesis that certain foods, beyond providing macronutrients, micronutrients, bioactive compounds, and energy, may also serve as sources of biologically active small RNAs. Although systemic uptake of these molecules remains under debate, dietary exposure to exogenous sRNAs has been documented [43,44,45,46]. Further research is needed to determine whether food-derived exRNAs can elicit functional effects in mammals.

In summary, our findings provide a deeper understanding of the sRNA cargo in human milk and offer compelling preliminary evidence that its unique properties may play a role in milk’s biological functions and vertical information transfer.

## 4. Materials and Methods

### 4.1. Donor Material, Collection, and Preparation Procedure

Sample collection was approved by the Ethical and Biosafety Committees of the School of Medical Sciences of Rosario National University (Resolution No. 3601/2022), and written informed consent was obtained from all donors following the Helsinki Declaration.

### 4.2. Blood Sample Collection

Serum samples were collected from healthy volunteers (n = 14; age range: 26–53 years). Venous blood was collected from healthy donors between 8 and 9 am and tubes were left upright at room temperature for 30–60 min to allow clotting, followed by centrifugation at 1300× *g* for 10 min. Samples were stored at −80 °C within 2 h of blood collection.

### 4.3. Human Milk Sample Collection

Human milk samples were collected from self-reported healthy lactating donors. Immediately before breastfeeding, donors collected milk using a sterile human pump into polypropylene tubes (50 mL, Falcon, Corning, NY, USA). The first 2–3 mL of milk was discarded to minimize potential contamination, and the subsequent 3–5 ml were collected for analysis (for colostrum samples, the full volume obtained was retained). Samples were transported on ice to the laboratory and processed within 1 h.

The primary screening cohort consisted of 8 donors of mature milk (3–6 months postpartum). In addition, an independent set of 9 samples spanning colostrum, transitional, and mature milk stages was analyzed. Donor demographic and clinical characteristics are provided in Supplementary Table S7.

### 4.4. Bovine Milk Sample Collection

Bovine milk samples (n = 13) were collected from Holando-Argentino cows raised under standard dairy farming practices on farms located in the Pampas region of Argentina (Santa Fe/Buenos Aires). This study was approved by the Institutional Committee for the Care and Use of Laboratory Animals and the Bioethics Committee of the School of Biochemical and Pharmaceutical Sciences of Rosario National University (No. 2022.00.40.317.622, Mat.Claus/Spinelli) and was carried out according to the ARRIVE guidelines. Collection was performed in the morning using aseptic techniques to prevent bacterial contamination. The udder was cleaned with an iodine-based disinfectant, and the first streams of milk were discarded before collecting the sample into sterile containers. Samples were transported on ice and processed within 2 h of collection.

### 4.5. Milk Sample Processing: Skim Milk Fraction Preparation

All milk samples were collected fresh, without pasteurization, and were processed immediately upon collection. To obtain the water-soluble fraction of the milk, known as skim milk (SM), samples were transferred to 15 mL conical centrifuge tubes and centrifuged at 2000× *g* for 10 min at 4 °C to remove cells. From this centrifugation, the intermediate phase was collected, ensuring that the top lipid layer was not removed when aspirating the underlying fraction.

Aliquots of the resulting cell-free supernatant were then transferred to Eppendorf tubes and centrifuged at 20,000× *g* for 1 h at 4 °C. After this centrifugation step, the intermediate phase was collected and sequentially filtered through 0.45 µm and 0.2 µm filters. Processed samples were stored at −80 °C for further analysis. All processing steps were carried out on ice.

### 4.6. RNA Sequencing

Total RNA samples were sent to Macrogen (Seoul, Republic of Korea) for library preparation and sequencing. Macrogen constructed the libraries using either the Illumina TruSeq Small RNA or Total RNA Library Preparation Kit (Illumina, Inc., San Diego, CA, USA), following the manufacturer’s protocols. The libraries were then sequenced on the Illumina NovaSeq platform, generating single-end 1 × 100 bp reads.

### 4.7. Selection of exRNA Datasets

We searched publicly available sRNA data with matching publications and sample metadata. Datasets were discarded if they presented samples without associated metadata, if there was a discrepancy in the number of samples reported, or if the raw sequencing data was unavailable. Raw sRNA sequencing data for 200 samples was downloaded from 9 serums and one mixed source dataset from the European Genome-Phenome Archive (EGA) and the Sequence Read Archive (SRA) with permission from the corresponding authors when required (GSE113994, GSE90028, GSE156874, GSE71579, GSE151963, GSE126051, GSE90524, GSE158312, GSE156693, EGAS00001003917 [3,47,48,49,50,51,52,53,54]). Only samples tagged as control or healthy patients in the metadata of the serum datasets were used. The dataset from Giraldez et al. [52] was split into the visualizations as two since they report two different techniques.

### 4.8. Bioinformatic Analyses

sRNA-seq data was processed using a custom pipeline built in Python 3.10. Raw sequencing data from human and bovine milk samples have been deposited in the NCBI Gene Expression Omnibus (GEO) under accession number GSE301917, with raw reads submitted to the Sequence Read Archive (SRA). Files were quality-controlled, following standard procedures. Briefly, sequencing adapters were trimmed using cutadapt (v. 3.5). Known contaminants were filtered out using the UNIVEC database and FastQC (v. 0.11.9) [55] was used for quality control. All sequences were then loaded into a MongoDB database (https://mongodb.com/), where further analysis occurred. Sequences were first aligned using bowtie2 (v. 2.4.4) [56] with a series of reference datasets which included human tRNA (GtRNAdb [57]), rRNA (Rfam [58], RNAcentral), miRNA (miRBase [59] and yRNA sequences among others. All remaining unknown sequences were aligned to the human genome (GRCh38) using HISAT2 (v. 2.2.1) [60]. Sequences were assigned using the corresponding feature file (GFF) using bedtools (v. 2.30.0). All statistics and visualizations related to sRNA-seq data were built by querying the database, building data frames in Pandas (v. 2.0.3) and visualizing with Plotly (v. 5.15.0). Sequence coverage and secondary structure plots were constructed using bowtie2, samtools (v. 1.13) [61] and VARNA (v. 3.93) [62]. The Python modules pysam (v. 0.21), pybedtools (v. 0.9) and varnapi (v. 1.0.0) were used. Pymongo (v. 4.4.0) and Mongoengine (v. 0.27.0) were used to connect to the MongoDB database. Reference sequences for known human sRNAs were obtained from curated databases. tRNA sequences were sourced from GtRNAdb (https://gtrnadb.ucsc.edu/), miRNA sequences from miRBase (http://mirbase.org/), and yRNA sequences from RNAcentral (https://rnacentral.org/). These references were used for mapping and annotation in our sRNA-seq analysis.

RNA-seq data was processed as follows. Reads were quality-checked (FastQC) and aligned to the GRCh38 reference genome with STAR. Differential expression analysis was carried out with DESeq2, using an adjusted *p*-value < 0.05 as threshold for significance.

### 4.9. Northern Blotting

RNA extraction from serum and milk samples was performed using Trizol (Invitrogen, Thermo Fisher Scientific, Waltham, MA, USA) according to manufacturer’s instructions. RNA samples were run on 10% TBE-urea polyacrylamide gels, transferred to positively charged nylon membranes (Roche, Basel, Switzerland). The membranes were cross-linked by UV irradiation. After cross-linking, the membranes were hybridized overnight at 40 °C with digoxigenin (DIG)-labeled DNA probes in DIG Easy Hyb solution (Roche, Basel, Switzerland). After low stringency washes (washing twice with 2× SSC/0.1% SDS at room temperature) and a high stringency wash (1× SSC/0.1% SDS at 40 °C), the membranes were blocked in blocking reagent for 30 min at room temperature, probed with alkaline phosphatase-labeled anti-digoxigenin antibody (Roche) for 30 min, and washed with 1× TBS-T. Signals were visualized with CDP-Star ready-to-use (Roche) and detected using Amersham Imager 600 (GE HealthCare, Chicago, IL, USA) according to the manufacturer’s instructions. Oligonucleotide probes were synthesized by Macrogen Inc (Seoul, Republic of Korea). DIG-labeled probes were prepared using the DIG Oligonucleotide tailing kit (2nd generation; Roche, Basel, Switzerland) according to the manufacturer’s instructions. The sequences of the probes were as follows: 5′ tDR-G (CTACCACTGAACCACCAATGC), 5′ tDR-E (TAACCACTAGACCACCAGGGA), 5′ tDR-V (TAACCACTACACTACGGAAAC), 5′ tDR-K (CTGATGCTCTACCGACTGAGCTATCCGGGC), 5′ yDR-4 (CCCACTACCATCGGACCAGCC), 5′ rDR-5.8S (CGCACGAGCCGAGTGATCCAC), 3′ rDR-5.8S (AGCGACGCTCAGACAGGCGTAGCCCCG), i rDR-5.8S (ACCCGGGGCCGCAAGTGCGTTCG), 5′ rDR-28S (CACGTCTGATCTGAGGTCGC), 5′ rDR-5S (GGCGCGTTCAGGGTGGTATGGCCGTAGAC).

### 4.10. RNA Decay Assays

To evaluate the stability of exogenous RNA in milk, we employed an assay previously optimized for comparative analyses of human biofluids [24]. This setup provides a functional readout of the degradative capacity of the biofluid, allowing comparison across independent replicates. Briefly, 1 μg heated and refolded U2-OS total RNA was added to 100 μL undiluted human skim milk sample and was incubated at 37 °C for variable periods (5, 15, 30 and 120 min). RNAs were purified by silica-based SPE using Monarch RNA Cleanup Kit (NEB, Ipswich, MA) and analyzed by northern blot.

### 4.11. RT-qPCR Assays

Levels of abundant milk exRNA were also analyzed by RT-qPCR. To determine the specific molecules to quantify by this technique we defined representative sequences based on sequence logos generated for milk exRNA fragments, prioritizing those with the highest relative abundance and consistent detection across different datasets (Supplementary Figure S7). RNA extraction from serum and milk samples was performed using Trizol (Invitrogen, Thermo Fisher Scientific, Waltham, MA, USA) according to the manufacturer’s instructions. sRNA levels were determined by stem-loop qRT-PCR as previously described [63,64]. cDNA was synthesized using Superscript III reverse transcriptase (Thermo Fisher Scientific, Waltham, MA, USA) and SLO-specific primers. PCR reactions were performed in a StepOne Real-Time PCR System (Thermo Fisher Scientific, Waltham, MA, USA) using HOT FIREpol EvaGreen Plus master mix (Solys Biodyne, Estonia) and specific forward and universal reverse primers. List of primers used in stem-loop RT q-PCR and q-PCR assays in Supplementary Table S8.

### 4.12. Extracellular Vesicle Isolation

Extracellular vesicles (EVs) were isolated from bovine skim milk using two complementary methods. First, EVs were isolated by ultracentrifugation as described by [65]. Briefly, 4 mL of different bovine skim milk samples were subjected to two consecutive ultracentrifugation steps at 100,000 *g* for 120 min at 4 °C to pellet EVs. The resulting pellets were washed with 1× PBS and resuspended in TRIzol reagent (Invitrogen) for subsequent RNA extraction. Additionally, 200 μL aliquots of the supernatants were collected and mixed with TRIzol reagent for RNA extraction.

For the second method, EVs were isolated by size exclusion chromatography (SEC). Column equilibration was performed at room temperature according to the manufacturer’s instructions. A 500 μL sample of bovine skim milk was applied to a washed qEV size exclusion chromatography column (qEV Original 70 nm, Izon Science Ltd., New Zealand), and 500 μL fractions were collected into 30 separate tubes using PBS as the eluent. Protein concentrations were determined using the Pierce™ BCA Protein Assay Kit (Thermo Scientific, Waltham, MA, USA) with bovine serum albumin (BSA) as the standard protein. Fractions rich in EVs (fractions 7 to 11), casein micelles (fractions 12 to 14), and extracellular molecules (fractions 15 to 26) were concentrated to a final volume of 250 μL using Vivaspin-6^®^ 10 kDa centrifugal concentrators (Sartorius, Hannover, Germany).

For RNA extraction and northern blot analysis, 200 μL of each concentrated fraction were mixed with TRIzol reagent. Additionally, 20 μL aliquots of each concentrated fraction were mixed with 1× Sample Buffer, separated by 15% SDS-PAGE electrophoresis, and stained with Coomassie Brilliant Blue to evaluate protein patterns and western blot analysis. Membranes were blocked with 5% BSA in PBS for 1 h at room temperature and incubated overnight with an anti-TSG101 antibody (1:500; Santa Cruz Biotechnology, Dallas, TX). Peroxidase-conjugated anti-mouse IgG (1:10,000; Jackson ImmunoResearch, West Grove, PA) was used as the secondary antibody. Immunoglobulins were detected using anti-goat secondary antibodies (1:10,000; Santa Cruz, Dallas, TX), owing to well-documented Fc-region conservation across ruminants. Chemiluminescence detection was performed using the Amersham ECL Prime Western Blotting Detection Reagent (GE Healthcare, Chicago, IL, USA).

### 4.13. Cell Culture

HEK239, HaCaT and Caco-2 cells were cultured in Dulbecco’s modified Eagle’s medium (DMEM) supplemented with 10% fetal bovine serum (FBS) and 100 units/mL penicillin and streptomycin (Invitrogen, Thermo Scientific, Waltham, MA, USA). HUVEC cells were cultured in RPMI 1640 medium supplemented with 15% FBS, 10 ng/mL VEGF (293-VE-010, R&D system) and 100 units/mL penicillin and streptomycin. THP1 cells were cultured in RPMI 1640 medium supplemented with 10% FBS and 100 units/mL penicillin and streptomycin. Cell lines were purchased from ATCC, and authenticity was documented by standard STR analysis. Cells were cultured in a humidified incubator at 37 °C with 5% CO_2_ and tested periodically for mycoplasma by 4,6-diamidino-2-phenylindole staining and PCR.

### 4.14. Viability Assays

Synthetic sRNA matching selected candidates (Supplementary Table S9) were purchased from Integrated DNA Technologies and dissolved in medium 0.1% FBS at 100 nM. The data presented in Supplementary Figure S9 shows that synthetic sRNAs do not exert cytotoxic effects over a wide range of concentrations.

Cell viability was analyzed using the MTT assay. Briefly, 24 h before treatment, cells were seeded in 96-well plates at a density between 7 × 10^3^ and 10 × 10^3^ cells per well depending on the cell line. As controls, cells were incubated with medium 0.1% FBS. For HUVEC cell viability sRNAs were dissolved at 100 nM in RPMI 0.1% FBS or in RPMI 0.1% FBS plus 10 ng/mL VEGF. As controls, cells were incubated with RPMI 0.1% FBS or RPMI 0.1% FBS plus 10 ng/mL VEGF. DNA oligonucleotides containing mainly identical sequence motifs were employed as additional controls: 5′ tDR-G DNA (GGCGGAGCATTGGTGGTTCAGTGGTAGAAT), 5′ tDR-V DNA (GGGTTTCCGTAGTGTAGTGGTTATCACG), 5′ tDR-E DNA (GGTCCCACATGGTCTAGCGGTTAGGATT), 5′ tDR-K DNA (GGCGGGCCCGGNTAGCTCAGTCGGTAGAGCA), 5′ yDR-4 DNA (GGCTGGTCCGATGGTAGTGGG), 5′ rDR-28S DNA (CGCGACCTCAGATCAGACG). After 24 h of treatment, cells were stained with 3-(4,5-dimethyl-2-thiazolyl)-2,5-diphenyl-2H-tetrazolium bromide (MTT) (0.5 mg/mL; Sigma) for 4 h at 37 °C. After the removal of the culture medium, formazan crystals were dissolved in DMSO and the absorbance was measured at 570 nm using a microplate reader.

### 4.15. Wound Healing Assay

HaCat cells were plated at high density and the following day a scratch was mechanically performed. After scratching, the medium was changed by 100 nM sRNA in DMEM 0.1% FBS or DMEM 0.1% FBS as control. Wound closure was monitored and imaged after 0, 2 or 24 h by inverted microscopy. At each time point, the number of cells per unit area was also counted in specific regions. Images were analyzed using ImageJ software (version 1.54f).

### 4.16. Statistical Analysis

Data are expressed as the mean ± s.e.m. and are representative of at least three experiments. Results were analyzed using Student’s *t*-test on two experimental groups, and ANOVA on three or more experimental groups. In all cases, *p*-values lower than 0.05 were considered statistically significant. All data were analyzed using GraphPad Prism 9.2.1 (San Diego, CA, USA) software.

## 5. Patents

The authors declare that a US Provisional Patent Application No. 63/734,414, “Bioactive milk-derived RNAs and associated methods”, has been filed related to the findings reported in this paper.

## Data Availability

The data underlying this article are available in the article, its online Supplementary Materials, and the Sequence Read Archive (SRA) at https://www.ncbi.nlm.nih.gov/geo/query/acc.cgi?acc=GSE301917 accessed on 12 December 2025.

## Supplementary Materials

The following supporting information can be downloaded at https://www.mdpi.com/article/10.3390/ncrna12010005/s1, Figure S1: Length of sRNA by type from sRNA experiments using human skim milk samples. Figure S2: Profile of abundant sRNA types across human milk lactation stages. Figure S3: Percentage distribution of the most abundant RNA fragments derived from tRNAs, rRNAs, and yRNAs i. Figure S4: exRNA profiling and quantification of Bovine skim milk. Figure S5: Comparative Analysis of sRNA Profiles in Human and Cow Milk. Figure S6: Purification of EV-rich fractions, casein micelle fractions, and extracellular medium fractions from bovine skim milk using qEV size exclusion chromatography. Figure S7: Sequence logos representing abundant tRNA, yRNA, and rRNA fragments. Figure S8: Effect of starvation and exRNA candidates on cell survival. Figure S9: Milk ex-RNA candidate (5´tDR-G, 5´tDR-E, 5´tDR-V, 5´tDR-K, 5´yDR-4 and 5’ rDR-28S) effect on cell viability over different cell lines. Table S1: sRNA biotype distributions in human milk sRNA-seq datasets (HM + HB) based on primary mapping assignments. Table S2: Relative abundance of tRNA-derived fragments based on primary mapping assignments. Table S3: Relative abundance of yRNA-derived fragments based on primary mapping assignments. Table S4: Relative abundance of miRNA based on primary mapping assignments. Table S5: Relative abundance of rRNA-derived fragments based on primary mapping assignments. Table S6: List of transcripts significantly altered by 5′ tDR-G in epithelial wound healing assays (RNA-seq, 2 h post-scratch). Table S7: Donor Demographic, Clinical, and Lactation Characteristics. Table S8: List of primers used in stem-loop RT q-PCR and q-PCR assays. Table S9: List of synthetic sRNA employed in in vitro assays.
