# Supplementary material for: Extracellular Small RNAs in Human Milk: Molecular Profiles, Stability and Fragment-Specific Responses in Cell-Based Assays
Source: Noncoding RNA. 2026 Feb 9;12(1):5. doi: 10.3390/ncrna12010005 (PMC12921927; doi:10.3390/ncrna12010005)
Supplement: Supplementary file 1 [file ncrna-12-00005-s001.zip › Suppl Figures_Claus2025-REVISED.pdf]

Supplementary Data

Extracellular Small RNAs in Human Milk: Molecular Profiles, Stability and Fragment-Specific Responses in Cell-Based Assays

Claus *et al*, 2026

Suppl. Fig. S1.

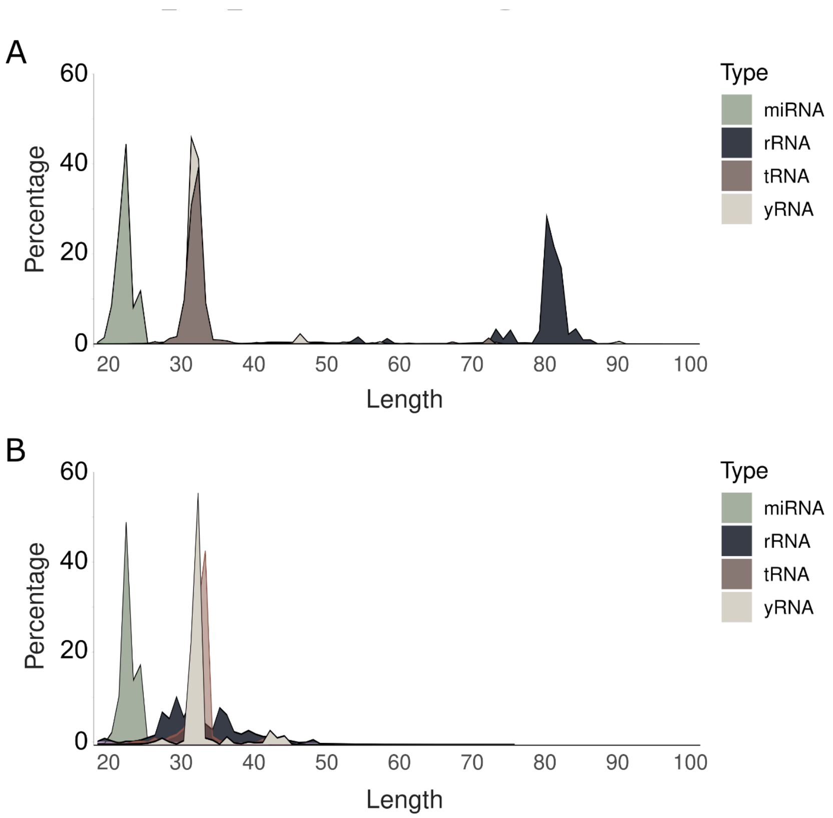

Supplementary Figure S1. Length of sRNA by type from sRNA experiments using human skim milk samples collected for this study **(A)** and those described in the Human Biofluid RNA Atlas **(B)**.

Suppl. Fig. S2.

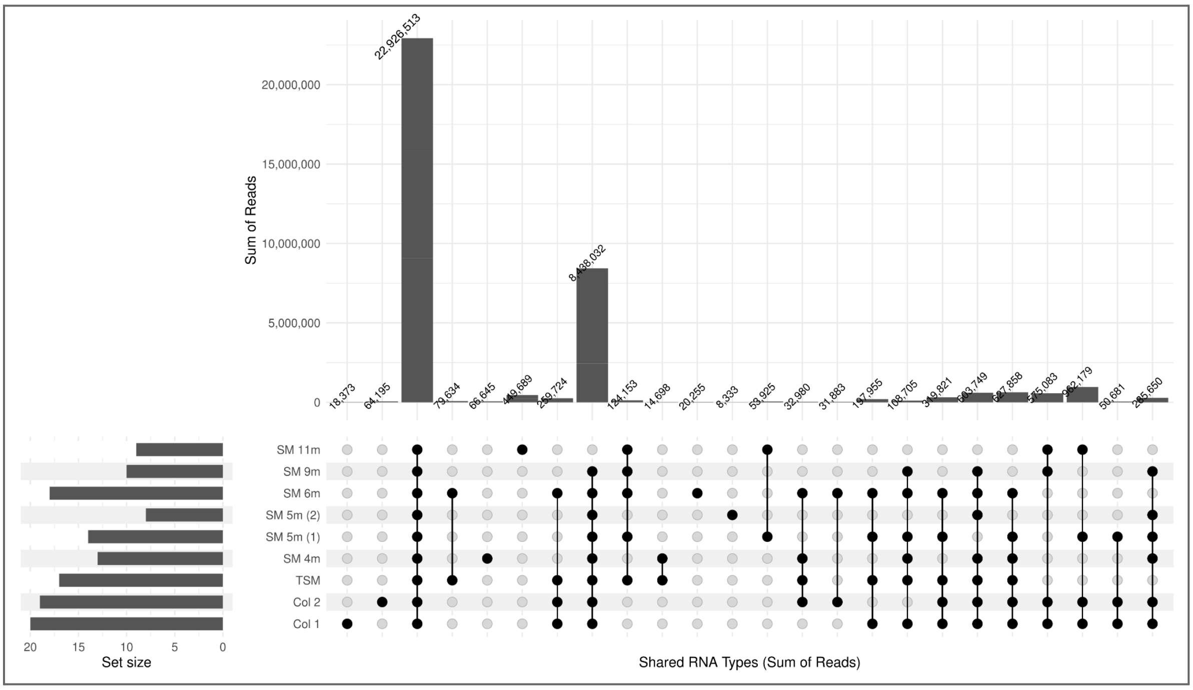

Supplementary Figure S2. Profile of abundant sRNA types across human milk lactation stages. Abundance-weighted Upset plot displaying the intersection of RNA types (primary assignments) across nine human milk samples. Vertical bars represent the cumulative read abundance (sum of reads) for RNA types shared by the datasets indicated with connected dots below. To focus on relevant cargo, only RNA categories representing >0.1% of total reads in any given sample were included. Sample abbreviations: Col, Colostrum; TSM, Transition Milk (5 days pospartum; SM, Mature Milk (m, months postpartum).

Suppl. Fig. S3

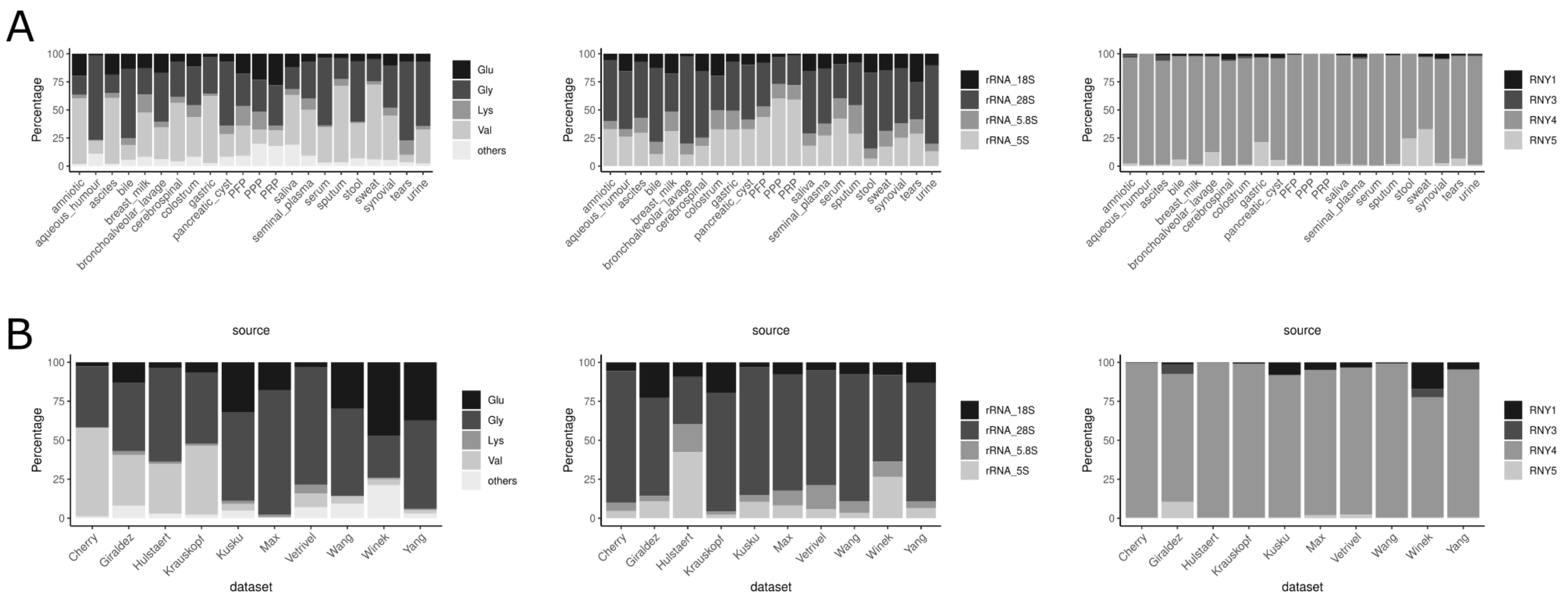

Supplementary Figure S3. Percentage distribution of the most abundant RNA fragments derived from tRNAs, rRNAs, and yRNAs in **(A)** 22 different human biofluids from the Human Biofluid RNA Atlas (Hulstaert et al., 2020); and **(B)** 10 independent sRNA-seq datasets from serum samples of healthy individuals. Categories with an average percentage of less than 5% across all samples were collapsed into the "others" category.

Suppl. Fig. S4.

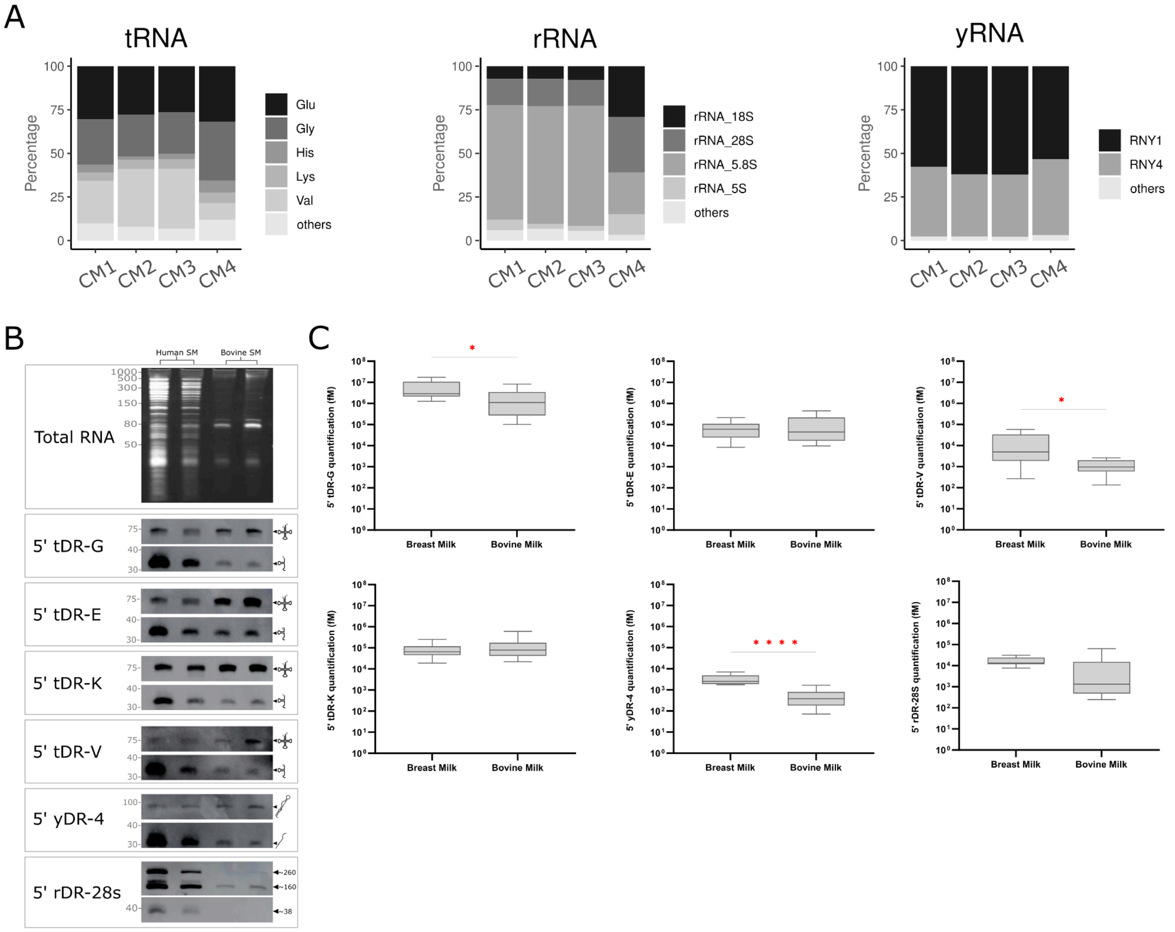

Supplementary Figure S4. exRNA profiling and quantification of Bovine skim milk. **(A)** Percentage distribution of the most abundant RNA fragments derived from tRNAs, rRNAs, and yRNAs in bovine skim milk. Categories with < 5% average abundance across all samples were grouped as “others”. **(B)** Northern blot of RNA samples purified from equal volumes (200uL) of human and bovine skim milk. Specific probes were used to detect the following RNA fragments: 5' tDR-G, 5' tDR-E, 5' tDR-V, 5' tDR-K, and 5' yDR-4. Exposure times of 3-4min were used. Abbreviations: PM, prestained marker; Bovine SM, skim milk; Human SM, skim milk. **(C)** Expression levels of 5′ tDR-G, 5′ tDR-E, 5′ tDR-V, 5′ tDR-K, 5′ yDR-4, 5′ rDR 28S, and 3′ rDR 5.8S were measured by SLO RT-qPCR in RNA from human (n = 8) and bovine (n = 13) skim milk . Data are shown as mean ± SEM. Quantification used standard curves from synthetic RNA oligos. Statistical analysis was determined using a two-tailed unpaired Mann–Whitney U test with α = 0.05; significance levels indicated as p<0.05 (*), p<0.001 (**), p<0.0001 (***).

Suppl. Fig. S5.

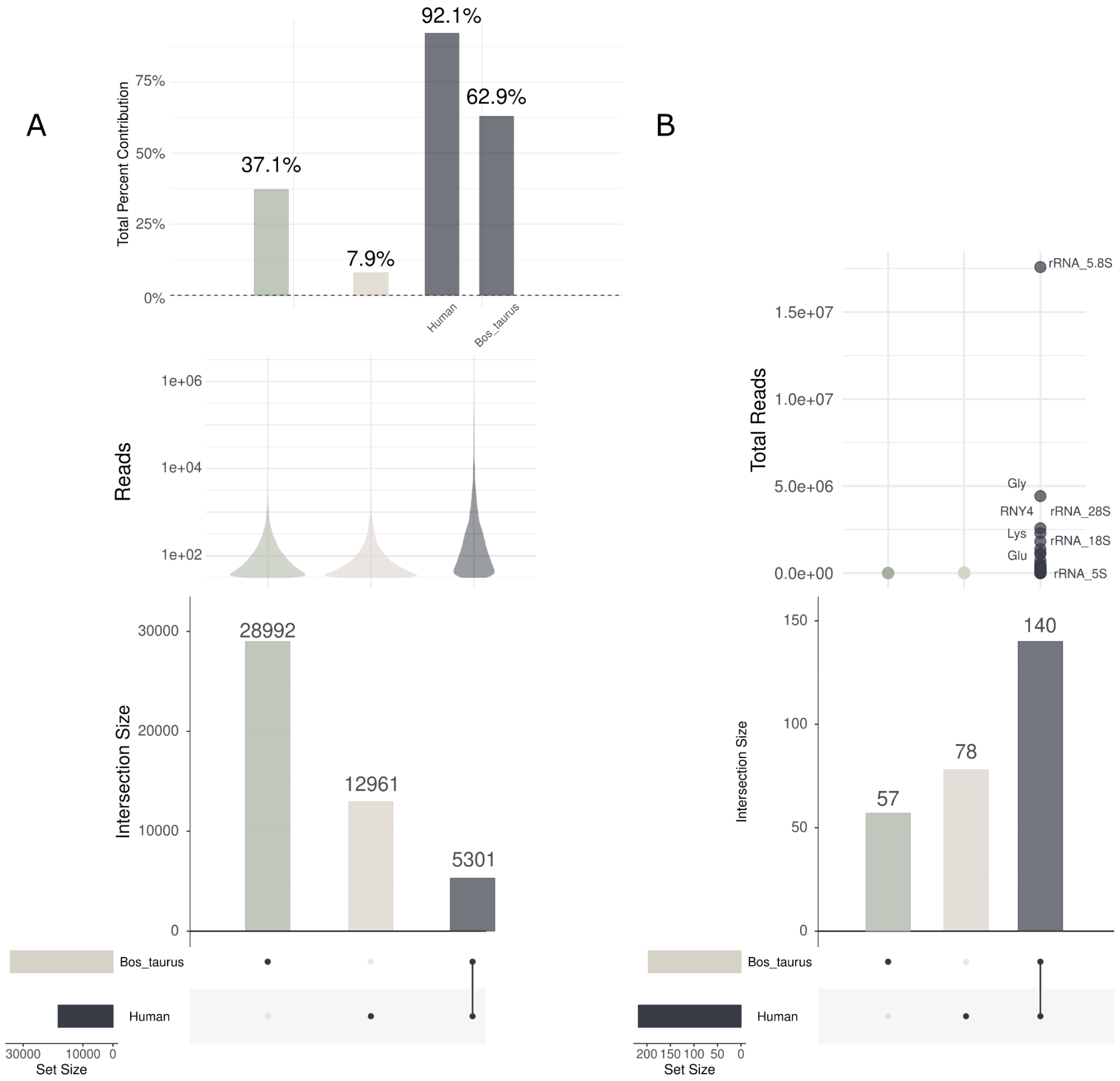

Supplementary Figure S5. Comparative Analysis of sRNA Profiles in Human and Cow Milk. **(A)** Overlap analysis of sRNA sequences. (Top) Bar plot showing the contribution of shared vs. species-specific sequences to the total read abundance within each species. When weighted by abundance, shared sequences constitute 92.1% of the total reads in human milk and 62.9% in bovine milk. The remaining fractions (37.1% in human and 7.9% in bovine milk) correspond to species-specific reads. (Middle) Violin plot depicting the log-transformed read count distribution for unique and shared sRNAs. (Bottom) UpSet plot showing that, despite the differences in abundance, 12,961 unique sRNA sequences are common to both species. **(B)** Characterization of the most abundant shared sRNAs. (Top) Scatter plot showing the total read counts for the most abundant RNA types within the shared fraction. Labels indicate the top 3% most abundant assignments. (Bottom) UpSet plot displaying the number of unique sequences for each of the main RNA families within the shared fraction.

Suppl. Fig. S6.

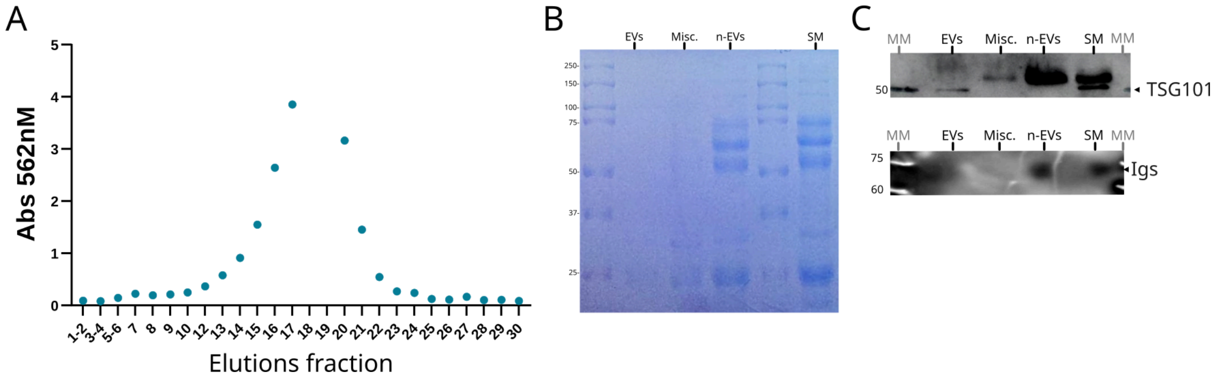

Supplementary Figure S6. Purification of EV-rich fractions (7–11), casein micelle fractions (12–14), and extracellular medium fractions (15–26) from bovine skim milk using qEV size exclusion chromatography. **(A)** Protein quantification of collected fractions. **(B)** 15% SDS-PAGE analysis followed by Coomassie staining, showing prominent casein bands in the micellar fraction. **(C)** Western blot analysis of TSG101, an established extracellular vesicle marker, and immunoglobulins (Igs), abundant proteins of the extravesicular compartment, demonstrating their selective enrichment in the corresponding fractions, consistent with successful isolation of EVs. skim milk; Misc, casein micelles.

Suppl. Fig. S7.

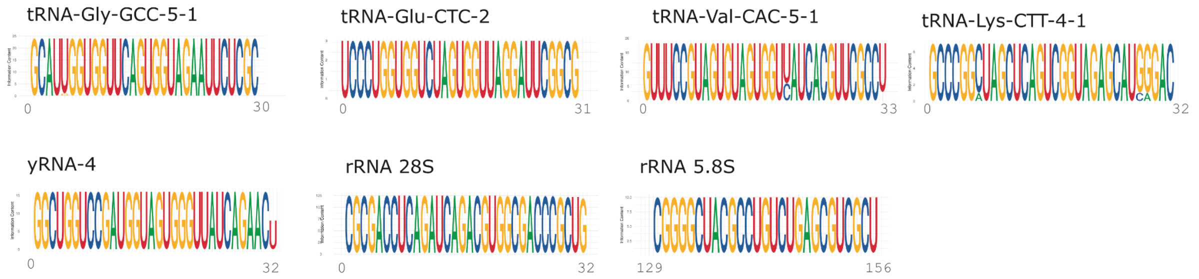

Supplementary Figure S7. Sequence logos representing abundant tRNA, yRNA, and rRNA fragments. The X-axis denotes the position in the sequence, while the Y-axis represents information content, as defined by original logo publication (Schneider & Stephens, 1990).

Suppl. Fig. S8.

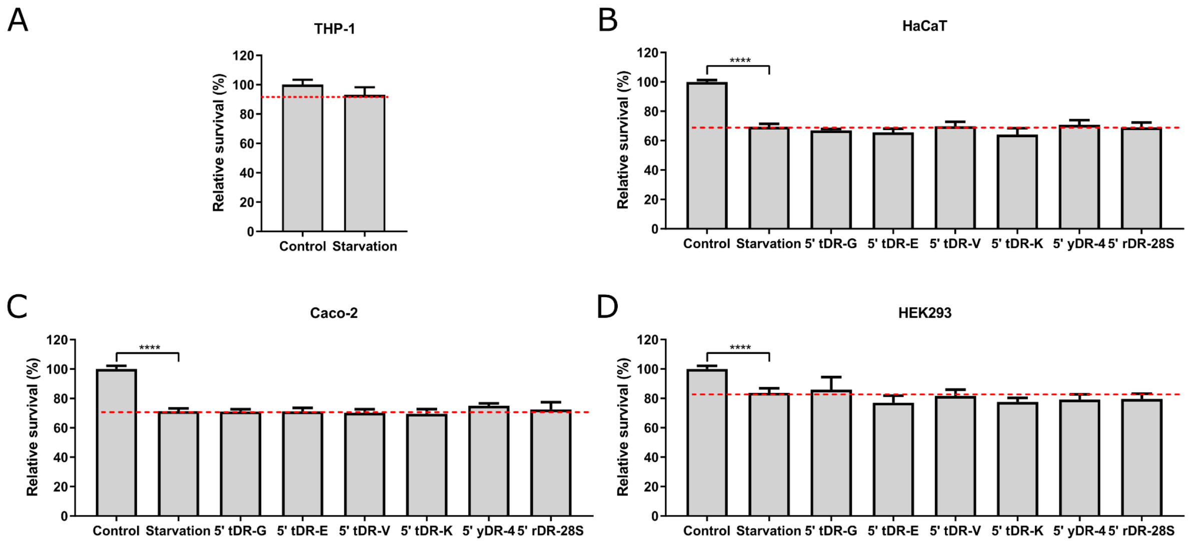

Supplementary Figure S8. Effect of starvation and exRNA candidates on cell survival. The tested exRNA candidates include 5’tDR-G, 5’tDR-E, 5’tDR-V, 5’tDR-K, 5’yDR-4, and 5’rDR-28S. Cell survival was evaluated under starvation conditions in four different cell lines: **(A)** THP-1, **(B)** HaCat, **(C)** Caco-2, and **(D)** HEK293. Statistical significance was assessed using a one-tailed t-test, with ****p < 0.0001.

Suppl. Fig. S9.

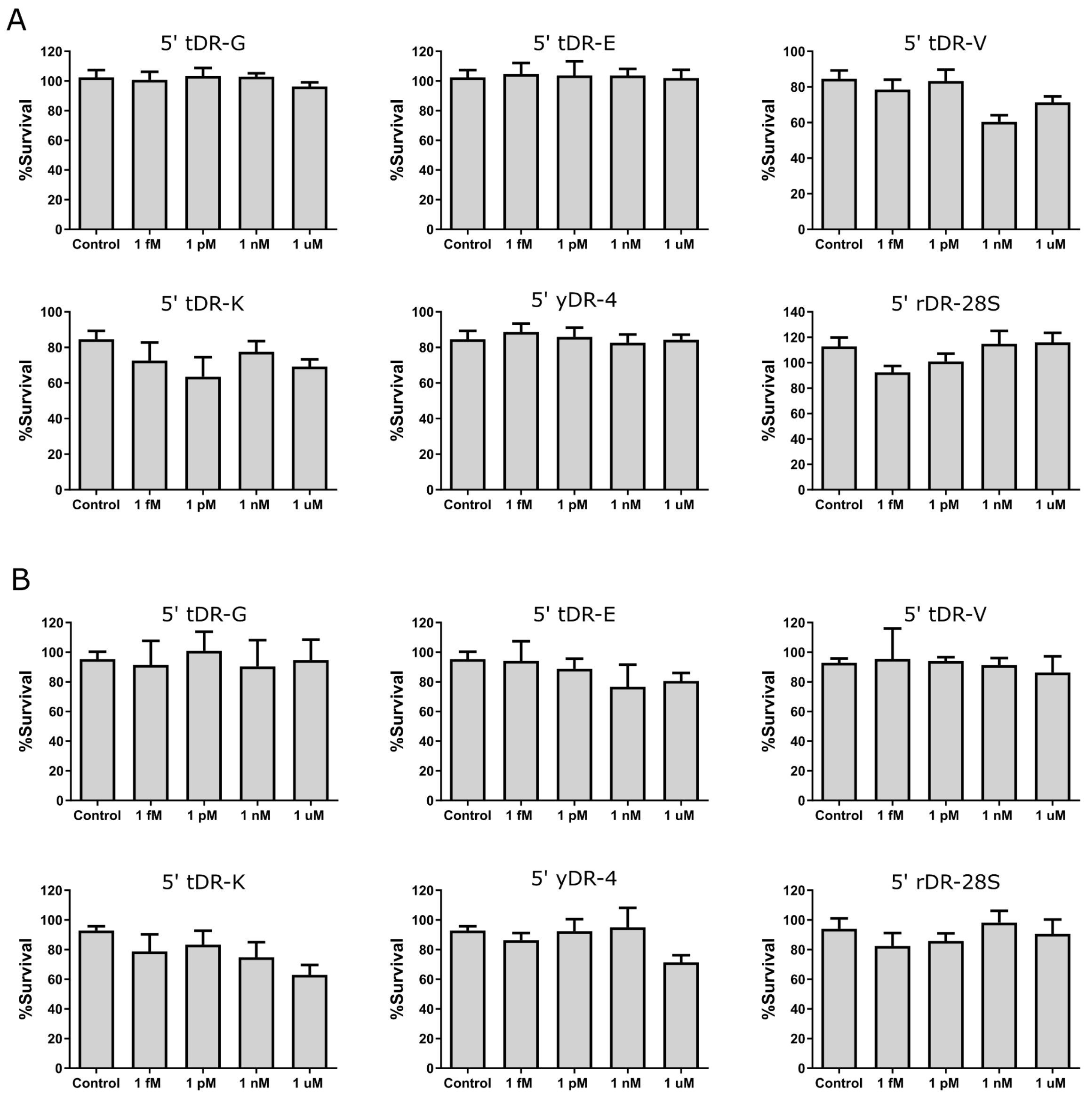

Supplementary Figure S9. Milk ex-RNA candidate (5´tDR-G, 5´tDR-E, 5´tDR-V, 5´tDR-K, 5´yDR-4 and 5’ rDR-28S) effect on cell viability over different cell lines. **(A)** Vero and **(B)** HEK293.
